# Adolescent pregnancy, public policies, and targeted programs in Latin America and the Caribbean: a systematic review

**DOI:** 10.26633/RPSP.2021.144

**Published:** 2021-12-16

**Authors:** Clara Rodríguez Ribas

**Affiliations:** 1 Universitat Pompeu Fabra Universitat Pompeu Fabra Barcelona Spain

**Keywords:** Pregnancy in adolescence, public policy, sexual and reproductive health, evidence-informed policy, Americas, Embarazo en adolescencia, política pública, salud sexual y reproductiva, política informada por la evidencia, Américas, Gravidez na adolescência, política pública, saúde sexual e reprodutiva, política informada por evidências, América

## Abstract

**Objective.:**

To present and assess evidence from Latin America and the Caribbean (LAC) on public policies and targeted programs which may have influenced variations in adolescent pregnancy or its proximate determinants, and to identify knowledge gaps that require further research.

**Methods.:**

A systematic review was performed based on the 2015 PRISMA protocol. Five databases were searched for articles published between 2000 and 2019 that refer to at least one country in LAC. The outcomes of interest were adolescent pregnancy or its proximate determinants (sexual behavior, contraceptive use, and/ or abortion). Only studies exploring correlations between the outcomes of interest and public policies or targeted programs were included in the analysis.

**Results.:**

Thirty studies spanning 14 countries were selected for analysis. Twenty-three of these (77%) were not included in prior systematic reviews on adolescent pregnancy. Public policies related to conditional cash transfers and compulsory education have the strongest evidence of correlation with adolescent pregnancy prevention. Emerging research points to the potential positive impact of life-skills programs for adolescents. Evidence from public health policies and programs was limited.

**Conclusions.:**

Further research which incorporates an intersectional analysis is needed to better understand which policies and programs could lead to steeper declines in adolescent pregnancy in the region. Evidence on effects of expanded family planning services and secondary school attainment upon adolescent pregnancy are particularly absent.

## RESUMEN

**Objetivo.:**

Presentar y evaluar evidencia de América Latina y el Caribe sobre las políticas públicas y los programas específicos que pueden haber influido en las variaciones en el embarazo en adolescentes o los factores determinantes relacionados, e identificar lagunas en el conocimiento que requieren más investigación.

**Métodos.:**

Se realizó una revisión sistemática de acuerdo con el protocolo PRISMA del 2015. Se llevaron a cabo búsquedas en cinco bases de datos en busca de artículos publicados entre el 2000 y el 2019 que se refirieran al menos a un país en América Latina y el Caribe. Los resultados de interés fueron embarazos en adolescentes o los factores determinantes relacionados (comportamiento sexual, uso de métodos anticonceptivos o aborto). Solo se incluyeron en el análisis estudios que exploraban las correlaciones entre los resultados de interés y las políticas públicas o programas específicos.

**Resultados.:**

Se seleccionaron para el análisis 30 estudios que abarcaban catorce países. Veintitrés de estos (77%) no estaban incluidos en revisiones sistemáticas anteriores sobre el embarazo en adolescentes. Las políticas públicas relacionadas con las transferencias monetarias condicionadas y la educación obligatoria arrojan la evidencia más fuerte de su correlación con la prevención de embarazos en la adolescencia. La investigación emergente indica una posible repercusión positiva de los programas para el desarrollo de aptitudes para la vida cotidiana dirigidos a adolescentes. La evidencia de las políticas de salud pública y los programas era limitada.

**Conclusiones.:**

Es necesario realizar investigaciones adicionales que incorporen un análisis interseccional para comprender mejor qué políticas y programas podrían conducir a un descenso más pronunciado del embarazo de adolescentes en la Región. La evidencia sobre los efectos de la ampliación de los servicios de planificación familiar y el nivel de estudios secundarios tras los embarazos en adolescentes es especialmente escasa.

## RESUMO

**Objetivo.:**

Apresentar e avaliar evidências da América Latina e do Caribe (ALC) sobre políticas públicas e programas direcionados que podem ter influenciado as variações na gravidez na adolescência ou em seus determinantes imediatos e identificar lacunas de conhecimento que requerem mais pesquisas.

**Métodos.:**

Foi realizada uma revisão sistemática com base no protocolo PRISMA de 2015. Buscas foram feitas em cinco bases de dados de artigos publicados entre 2000 e 2019 que se referem a pelo menos um país da ALC. Os resultados de interesse foram a gravidez na adolescência ou seus determinantes imediatos (comportamento sexual, uso de métodos anticoncepcionais e/ou aborto). Somente os estudos que analisavam as correlações entre os resultados de interesse e políticas públicas ou programas direcionados foram incluídos nesta análise.

**Resultados.:**

Trinta estudos englobando 14 países foram selecionados para a análise. Vinte e três deles (77%) não foram incluídos em revisões sistemáticas prévias sobre gravidez na adolescência. As políticas públicas relacionadas a transferências monetárias condicionais e à educação obrigatória têm as evidências mais sólidas de correlação com a prevenção de gravidez na adolescência. Pesquisas recentes apontam para o potencial impacto positivo de programas de habilidades para a vida para adolescentes. As evidências de políticas e programas de saúde pública foram limitadas.

**Conclusões.:**

São necessárias mais pesquisas que incorporem uma análise cruzada para entender melhor quais políticas e programas levariam a uma maior diminuição da gravidez na adolescência na região. Particularmente, faltam evidências sobre os efeitos dos serviços ampliados de planejamento familiar e da conclusão do ensino médio na gravidez na adolescência.

In Latin America and the Caribbean (LAC), adolescents aged 15–19 account for 16.0 percent of total fertility among women of reproductive age (2015–2020), which represents the highest share of adolescent pregnancy of any region in the world (1). An increasing proportion of these pregnancies is unintended, particularly among lower poverty quintiles (2). These trends respond to a variety of deprivations including high unmet-need for contraceptives among adolescents, absence of opportunities, and deeply rooted gender inequalities among others (3,4). Adolescent fertility can pose severe challenges to an individual’s development and is strongly associated with school dropout, lost productivity, the intergenerational transmission of poverty, and high rates of maternal mortality and morbidity (4).

As indicated by the 2013 Montevideo Consensus and periodic reviews of its implementation, governments in the region have been making continuous progress in addressing priorities around gender equality, sexual and reproductive health, and the specific needs of young people (3). Despite these investments, adolescent pregnancy reduction has been slow and uneven across and within countries, especially when compared with other parts of the world (2). Moreover, “the shortage of systematic documentation and research on the issue in the [LAC] region makes it difficult to understand why these efforts have not generated better results” (5).

To understand adolescent fertility, one must take into consideration that not all pregnancies come to term, due to both natural causes and voluntary interruptions. Moreover, not all sexual encounters result in pregnancy. However, sexual behaviors can provide some insight into the risk of adolescent pregnancy occurring. As a result, adolescent sexual behaviors, which include early sexual initiation, sexual activity, and contraceptive prevalence, serve as proxies to measure the risk of pregnancy exposure. The academic literature considers these behaviors to be proximate determinants of adolescent pregnancy and employs them to study and measure the effects of policies and programs (6).

This systematic review addresses the knowledge gap in the intended and unintended effects that public policies in LAC countries have on adolescent pregnancy. It aims to present and assess key evidence from the region about what policies have been shown to correlate with variations in adolescent pregnancy through a thorough appraisal of peer-reviewed articles studying relationships between public policies or targeted programs and adolescent pregnancy. By presenting the collective evidence available from the region, this review also aims to identify areas that require further exploration, which ultimately will inform how public policies and programs can prevent unintended adolescent pregnancy.

## MATERIAL AND METHODS

Global evidence suggests that reducing unintended adolescent pregnancy is best addressed through multidisciplinary approaches (4,7). Therefore, this review adopted a search strategy that identified both public policies and targeted programs, exploring their (un)intended effects on adolescent pregnancy and its proximate determinants (contraceptive use, sexual behavior, and/or abortion). The scope of this systematic review is broader than prior reviews: it includes articles in multiple languages and from across policy sectors, and it explores relationships across multiple relevant outcomes of interest (8–16).

The database search included terms in both English and Spanish,^1^ which comprised: (i) descriptions of adolescents (e.g. (*adolescent* OR *teenage); (adolescente)*); (ii) descriptions of pregnancy and its related proximate determinants (e.g.[*motherhood* OR *pregnancy* OR *fertility* OR *birth rate* OR *childbearing*] AND/OR [*prevention* OR *reduction*] AND/OR [*abortion* OR *behavior* OR contracept*]; [*maternidad* AND (*temprana* OR *precoz*)] AND/OR [*prevención* OR *reducción*] AND/OR [((*iniciación*) OR (*primera*) OR (*conducta*) AND (*sexual*)) AND/OR (*anticoncepción*) AND/OR (*planificación familiar*) AND/OR (*aborto*)]; (iii) [(public polic*) AND/OR (program*)]; [(política* pública*) AND/OR programa*]; and (iv) references to LAC (e.g.*Latin America and the Caribbean* OR [*country name list*]).

The searches were conducted in EBSCO, The Cochrane Library, PubMed, LILACS, and The World Bank JOLIS. The final search was conducted in September 2019.

*A priori* inclusion criteria were: (i) case study included a LAC country; (ii) outcome variables included behavioral effects or adolescent pregnancy; and (iii) effects were measured on adolescents (youth aged 10 to 19).^2^

To focus on evidence from policies responding to the updated sexual and reproductive health paradigm contemplated in the ICPD Plan of Action and reflected in the Millennium Development Goals, articles were excluded if they presented only changes in knowledge and attitudes without assessing changes in behavior, or if they were published prior to 2000.

To confirm exclusion/inclusion, the review manually screened article titles and, when these did not provide sufficient information, abstracts. The review identified articles through both search results and a snowballing exercise of references. A spreadsheet (Microsoft Office Excel v16.49) of all results tracked information on whether the article was included/excluded following review of the title, abstract, or full paper, with key words describing the rationale for exclusion. The search, identification, screening and eligibility analysis followed the 2015 PRISMA guidelines (17).

## RESULTS

**FIGURE 1.**
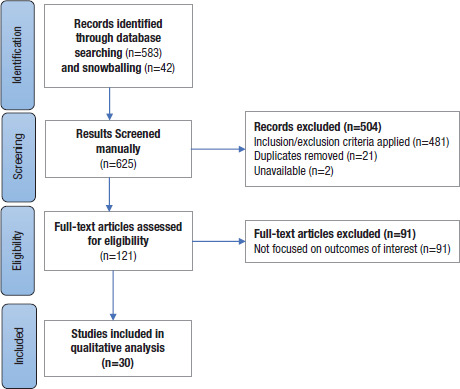
Search and screening process

***Source*:** based on the results of the present study.

The search and screening process, presented in Figure 1, rendered 30 articles for inclusion in this review, the details of which are presented in Table 1. Quality was reviewed following STROBE guidelines, and only those with a 80% score or more were retained. The vast majority of articles (77%, n=23) have not been included in prior relevant systematic reviews (Table 2). A thematic analysis of results yielded four policy or program sectors in which to group articles: conditional cash transfers, education, health, and life skills.

### Conditional cash transfers

Nine relevant studies explored the relationship between conditional cash transfer (CCT) policies and adolescent pregnancy or its proxy indicators.

Several studies in this category examined the policy *Progresa*/*Oportunidades,* implemented in Mexico.^3^ This CCT entails the transfer of a monthly stipend to families, subject to conditions related to school attendance, health talks and check-ups, and nutrition. Research found effects vary across rural and urban populations, as well as during different stages of the program.

A few studies found positive effects of *Oportunidades* on adolescent pregnancy or proximate determinants. One study focusing on urban populations across Mexico found delayed motherhood and age of sexual initiation as a result of exposure to CCT (18). Another study found an increase in contraceptive use among beneficiaries, with effects varying in degree according to poverty level in rural areas (19). A larger effect was found among the poorest women, which was interpreted to result from the program’s health talks having a stronger impact on those who were originally most marginalized and had lower contraception knowledge, coupled with the poorest beneficiaries’ higher compliance in attending the talks to ensure continued financial allocations. Other studies conducted on the effect of *Bolsa Familia*, a CCT implemented in Brazil, and *Juntos*, in Peru, also found participation in the programs to be negatively associated with adolescent pregnancy (20–22).

**TABLE 1.** Characteristics of included articles, by intervention type (n=30)

Country Case Study (Year of publication)	Outcome of interest	Policy/Intervention Result	Reference
**Conditional cash transfers (n=9)**			
Mexico (2009)	Age at first sex (urban)	+ (reduction observed)	(18)
Mexico (2010)	Contraceptive use	+ (larger effect among poorest, smaller effect closer to threshold)	(19)
Brazil (2012)	Adolescent fertility	+ (reduction observed)	(20)
Peru (2012)	Adolescent fertility	+ (reduction observed)	(21)
Brazil (2019)	Adolescent fertility	+ (reduction observed)	(22)
Mexico (2015)	Adolescent pregnancy (rural)	no effect	(23)
Mexico (2013)	Adolescent pregnancy (rural)	no effect	(24)
Colombia (2018)	Adolescent pregnancy	+ (with stronger effect in CCT with performance conditionality)	(25)
Honduras (2007)	Adolescent fertility	- (increase observed)	(26)
Mexico (2007)	Adolescent fertility	no effect	(26)
Nicaragua (2007)	Adolescent fertility	no effect	(26)
**Education (n=14)**			
Chile (2011)	Adolescent pregnancy	+ (stronger reduction among poor and urban)	(27)
Peru (2019)	Adolescent pregnancy	+ (reduction observed)	(28)
Argentina (2017)	Adolescent fertility	+ (reduction observed)	(29)
Chile (2005)	Adolescent pregnancy	+ (reduction observed)	(35)
Chile (2012)	Sexual behavior (urban)	no effect	(30)
Mexico (2008)	Sexual behavior (urban)	no effect	(31)
Jamaica (2007)	(i) Age at first sex; (ii) Use of modern contraception at first sex	(i) no effect; (ii) + (short term effect)	(32)
Mexico (2006)	Condom use	no effect	(33)
Brazil (2001)	Contraceptive use (urban)	no effect	(34)
	Sexual behavior	no effect	
Belize (2004)	Condom use	+	(36)
Mexico (2004)	Condom use	+	(37)
Haiti (2015)	Adolescent pregnancy	+ (stronger reduction effect in integrated intervention)	(38)
Mexico (2019)	Use of modern contraception at first sex	+	(39)
Brazil (2009)	(i) Age at first sex; (ii) Use of modern contraception in last sex	(i) no effect; (ii) + (stronger effect among older adolescents)	(40)
**Health (n=5)**			
Colombia (2008)	Adolescent pregnancy	+ (reduction observed but non-attribution to policy)	(41)
Mexico (2016)	Adolescent pregnancy	no effect	(42)
Uruguay (2018)	Adolescent pregnancy	no effect	(43)
Uruguay (2018)	Adolescent pregnancy	+ (reduction observed)	(44)
Paraguay (2018)	Adolescent pregnancy	+ (reduction observed but non-attribution to policy)	(45)
**Life-Skills (n=2)**			
Dominican Republic (2014)	Adolescent pregnancy	+ (reduction observed)	(46)
Dominican Republic (2016)	Adolescent pregnancy	+ (reduction observed)	(47)

***Source*:** based on the results of the present study.

**TABLE 2.** Published systematic reviews exploring relationship between public policy interventions and adolescent pregnancy or its proxy indicators, 2000-2019

Author (Year) (reference)	# Studies reviewed^1^	Intervention	Outcome(s) of interest	Key Inclusion criteria	Case studies also included in this review (n=7)
**Sanz-Martos et al., (2018)** (8)	24	Educational interventions	Pregnancy prevention, sexual behavior, contraceptive use	No behavior change measurement	(35)
**Salam et al., (2016)** (15)	51	Adolescent SRH program (educational interventions, pregnancy prevention)	Contraceptive use, sexual behavior, pregnancy	Randomised, Quasi-randomised and before/after studies	(33)
**Hindin et al., (2016)** (9)	21	SRH interventions	Pregnancy prevention; sexual behaviour, contraceptive use	Low-and Middle-Income countries	(18)
**Lopez et al., (2016)** (10)	11	School-based interventions	Contraceptive use	Random Control Trials	(33)
**Oringanje et al., (2010)** (11)	41	Primary prevention interventions (school-based, community/home-based, clinic-based, and faith-based)	Risk of unintended pregnancy; sexual behavior, contraceptive use, abortion	Individual and cluster randomised control trials (RCTs)	(31, 33, 35)
**Speizer et.al., (2003)** (12)	41	Young adult SRH programs on knowledge, attitudes and behaviors	SRH knowledge, attitudes and behavior	Experimental or quasi experimental designs	(34)
**Gottschalk and Ortayli, (2014)** (16)	15	Contraceptive services and interventions	Contraceptive use	Low-Middle Income Country	(31, 34, 40)
**Kirby et.al., (2007)** (13)	83	Curriculum-based sex and HIV education programs	Sexual behavior	Experimental or quasi-experimental design	(35, 36)
**Denno et.al., (2015)** (14)	36	SRH interventions (in/out facilities)	Utilization of services; STI/HIV/and pregnancy rates	Low-and Middle-Income countries; English	(34)

1 Although some reviews included more studies from LAC, several were excluded from the current review according to the inclusion and exclusion criteria. Several studies were included in more than one systematic review.

SRH, sexual and reproductive health

***Source*:** based on the results of the present study

Nevertheless, studies focusing on a later period of implementation of the CCT in Mexico found that while *Oportunidades* did not have a direct effect on adolescent pregnancy rates among beneficiaries and that increased educational attainment, marriage, and pregnancy experience were associated with increased contraceptive prevalence rate with no relationship with exposure to the program (23,24).

Another set of comparative studies explored whether the conditions established within the CCT programs can explain the variations in adolescent pregnancy. One of these studies explored the impact of different conditions of CCTs implemented in Bogotá, Colombia (25), where one policy conditioned certain benefits to the completion of high school while the other did not. Using a representative survey, the study authors found that school performance requirements had a positive effect on reduced teenage pregnancy, versus attendance requirements alone, which had no effect. They concluded that conditions on academic performance provide greater incentives to avoid or delay pregnancy, and that delaying the rewards of the programs reduces these incentives.

A cross-country comparative analysis found that in Mexico (prior to 2009) and Nicaragua, where CCT programs did not adjust financial allocations for increased number of children, no increase in adolescent fertility was identified (26). However, in Honduras CCT conditions appear to have created large fertility incentives, although only among older age cohorts, beyond adolescence. The study authors propose that the substantial increase in financial transfers received in the Honduras case offset the opportunity cost of having another child, thereby generating the incentive.

### Education

The review identified 14 papers exploring the effect of education-related policies on adolescent pregnancy. Three of them investigate educational reforms affecting the whole population, while 11 others explore the impact of specific curricula taught in controlled settings with the objective of changing knowledge, attitudes, and behaviors related to adolescent sexual and reproductive health.

In the case of Chile and Peru, changes in education policies extended compulsory school hours (27,28). Evidence points to an inverse correlation between implementation of these reforms and adolescent pregnancy rates, with the effect being stronger among poor urban adolescents in Chile. In Argentina, where a reform increased compulsory years of schooling from 7 to 10, a study found a strong correlation between additional school years and increased enrolment rates following the new policy and reduced adolescent pregnancy rates (29).

Five studies exploring the effect of sexual and reproductive health (SRH) sessions imparted in targeted high-schools in Brazil, Chile, Jamaica, and Mexico found no evidence of lasting behavioral changes on adolescents as a result of the interventions (30–34). In the case of Jamaica, the study found no effect on sexual activity, but a positive one on contraceptive use at first intercourse—although this effect did not last (32).

An additional study from Chile, using a random-control trial to explore the long-term effect of an abstinence-centered curriculum, found higher pregnancy rates among adolescents not exposed to the sex-education program (35). Two other small-sample studies from Belize and Mexico exploring the effect of knowledge sessions imparted to high school adolescents found positive effects on condom use, although the Mexican study found no effect on reported sexual practices or unprotected sexual intercourse (36,37). Lastly, one study conducted in Haiti compared the results of a stand-alone SRH curriculum intervention with one that combined SRH sessions with sport activities (38). While both interventions showed positive effects on reducing adolescent pregnancy among participants compared to control groups in the same village, a stronger effect was found in the integrated approach than in the stand-alone SRH education sessions.

A few studies explored the effect of larger sexuality education programs. Evidence indicates adolescents who had received sexuality education in Mexico had higher chances of using modern contraception at first intercourse (39). A study on the adolescent population in selected high schools in Minas Gerais municipalities, Brazil, found that participation in sexuality education courses doubled consistent use of condoms with casual partners and substantively increased the use of modern contraceptives during last intercourse (40). However, participation appeared to have no effect on age at first intercourse nor on adolescents’ engagement in sexual activities.

### Health

Five studies explored the impact of health policies related to service delivery, access to abortion, and access to contraception and HIV prevention.

A study on the impact of the Colombian SRH policy implemented from 2002 to 2006 found an uneven decrease in adolescent pregnancy rates across locations but could not confirm national policy as the driver (41). Notably, this is the only study identified in this review which attempted to measure the impact of a national health policy on adolescent pregnancy.

Three studies explored the effect of abortion legalization on adolescent pregnancy. They found legalization did not reduce childbearing among adolescents in the three years after its adoption in Mexico City (42), although the findings are mixed in Uruguay (43,44). Comparing 2010 with 2014 adolescent fertility data from health centers in Montevideo one study found no effect of abortion legalization upon adolescent pregnancy nor fertility rates (43). However, a later study comparing national data from 2006 and 2012, found access to legal abortion correlated with reduced adolescent fertility (44).

A final study from Paraguay seeked to identify the effect of expanded health coverage, through the introduction of outreach first-level clinics, on adolescent pregnancy (45). The study found a significant correlation between the establishment of these clinics and a reduction in adolescent pregnancy but was unable to determine causality.

### Life skills

The search identified two studies on the effect of the skills-based program *Juventud y Empleo* implemented in Dominican Republic, which targeted youth who were out of school and most at risk. The program combined classroom training and internship placements, and integrated socio-emotional skills building. An impact evaluation of the program found that *Juventud y Empleo* reduced the probability of teenage pregnancy in the treatment group, with a stronger effect on young and single women and those who were already mothers (46,47). The study authors attributed this effect to an overall increase in youth expectations about the future, resulting from the program. However, no fertility effect was found on older cohorts (20–24), which may indicate the importance of providing alternative expectations for the future early to impact fertility patterns.

## DISCUSSION

Results from this review point to a variety of pathways through which public policies may have (un)intended effects on adolescent pregnancy and its proximate determinants.

The analysis supports evidence that CCTs have significant effects on decisions about motherhood. The majority of studies of CCTs find that financial transfers conditioned to health and education requirements have a positive effect on adolescent pregnancy prevention. While the studies cannot always attribute positive changes in behavior or reductions in adolescent pregnancy to these policies, there is no evidence that these policies are detrimental to positive changes in behavior or reductions in adolescent pregnancy, with one exception, Honduras, where the CCT benefits for higher fertility rates offset its opportunity cost.

Research from LAC appears to confirm an incapacitation effect both of education, which reduces engagement in risky sexual behavior, and of the human capital effect, whereby education generates an increase in the opportunity cost of adolescent pregnancy versus investing in future opportunities (48). This evidence is supported by research from other regions, whereby mandating women to remain in school longer significantly reduces the likelihood of motherhood in adolescence (49). Studies theorize that the mechanisms involved include increased exposure to sexual and reproductive health information, an ability to make informed choices about when and if to have sex, and a result of modified aspirations as alternatives to adolescent motherhood are presented (50).

The studies reviewed show limited evidence for the effectiveness of behavior-centered interventions in modifying risky sexual behavior. This mirrors findings from other regions, which also present mixed and often inconclusive results (11). Evidence indicates behavioral change is sustained when accompanied by normative changes, thus requiring a broader set of interventions beyond imparting stand-alone programs (4)*.* Predictably, then, the studies reviewed found behavioral effects in more innovative approaches, such as Haiti’s combination of SRH education with sports, or Chile’s health referrals. In addition, evidence from Brazil and Mexico supports the hypothesis that comprehensive sexuality-education does not accelerate sexual initiation.

The studies found on the relationships between health policies and adolescent pregnancy in LAC are limited in number and diverse in scope, ranging from assessments of national health policies to smaller, localized interventions, making it difficult to draw conclusions. Although results remain inconclusive and merit further research, the three studies on the effects of abortion legalization offer an important contribution to the global literature.

Good health enables young people to attend school and increases academic performance, as well as labor market insertion. The evidence reviewed in this study on the effect of health sector policies on adolescent fertility is limited but promising. Investments in health are also associated with closer contact with health professionals, who may provide SRH information and services, expanding the fertility choices available to women and girls. This seems particularly relevant in LAC, where estimates indicate adolescent pregnancy would be reduced by 43% if the unmet need for family planning was satisfied (51).

The original research on the youth livelihoods program in the Dominican Republic indicates that modifications in young people’s aspirations may affect fertility choices. The studies reviewed here, together with nascent literature on empowerment and livelihood for adolescents programs implemented in other regions, point to the potential of this type of policies (52). Research from LAC has shown that opportunities—or lack thereof, as illustrated by high youth unemployment—are important factors in explaining the persistently high rates of adolescent pregnancy (4). If young people’s aspirations for their future shape pregnancy decisions, evidence indicates there is a greater incentive to delay parenthood when this future includes an opportunity for employment and its corresponding income.

As a whole, the evidence presented in this review largely echoes global guidance about the combination of policies and programs that may prevent adolescent pregnancy (53). These pathways, which may originate in the education, health, life-skills, and poverty alleviation sectors, are a reflection of the multidisciplinary approaches that scholars argue are required to address adolescent pregnancy (4). They are also a reflection of the intrinsic complexity of challenges posed by adolescent pregnancy, as well as of the multiplicity of factors which act as drivers of fertility.

The analysis encountered two main limitations. The first is the vast heterogeneity of methodologies, interventions, sample sizes, and outcomes across studies, derived from the broad search conducted, which precluded the possibility of conducting a meta-analysis and of generalizing evidence. Important differences across LAC countries, given their varied population and geographical size, culture, ethnic and racial diversity, as well as state administrative organization, among other characteristics, also complicates generalizability. The second limitation of the evidence collected is its narrow disaggregation by sex and age, as well as by other intersectional characteristics. Consequently, there is little evidence on the differential effects of policies between younger and older adolescents, rural versus urban, and among indigenous populations. This is particularly relevant in a region where inequalities and poverty are closely linked to geographical area of residence and ethnicity/race, as is the incidence of adolescent pregnancy.

Emerging evidence on the ability of life-skills policies to modify youth aspirations is promising and merits further investigation (4). Equally important is the need to untangle the differential effects of primary versus secondary education: high levels of primary school enrollment and completion have already been achieved across countries, but better understanding of the incapacitation effects of secondary school attainment is needed (54).

Given that adolescents’ unmet need for contraceptives has been highlighted as one of the key drivers of pregnancy among girls aged 15–19, especially in LAC, it is surprising that this review found no studies on this subject. Important gaps persist, with more studies needed on health policies targeting adolescents.

## Disclaimer.

Author hold sole responsibility for the views expressed in the manuscript, which may not necessarily reflect the opinion or policy of the RPSP/ PAJPH and/or PAHO.

## Footnotes

1 Some search results in these languages led to articles in Portuguese, which the review considered if they fulfilled the inclusion criteria.

2 Some studies included this age group and older cohorts. In these cases, the review included the studies as the findings are relevant to the object of study.

3 The program began in 1997 as Progresa (Programa de Educación, Salud y Alimentación) and was renamed Oportunidades in 2001.
